# Infections in Inborn Errors of STATs

**DOI:** 10.3390/pathogens13110955

**Published:** 2024-11-01

**Authors:** Chen Wang, Alexandra F. Freeman

**Affiliations:** Laboratory of Clinical Immunology and Microbiology, National Institute of Allergy and Infectious Diseases, National Institutes of Health, Bethesda, MD 20892, USA; chen.wang@nih.gov

**Keywords:** STAT, pathogen, antimicrobials, hematopoietic cell transplantation

## Abstract

The Janus kinase (JAK)-signal transducer and activator of transcription (STAT) pathway is highly conserved and essential for numerous biological functions triggered by extracellular signals, including cell proliferation, metabolism, immune response, and inflammation. Defects in STATs, either loss-of-function or gain-of-function defects, lead to a broad spectrum of clinical phenotypes in humans, including a wide range of infectious complications. The susceptibility to pathogens can stem from defects in immune cells within the hematopoietic compartment, impaired barrier functions of non-hematopoietic compartment, or a combination of both, depending on the specific STAT defect as well as the pathogen exposure history. Effective management involves antimicrobial prophylaxis tailored to the patient’s infection risk and improving disease control with targeted therapies and/or hematopoietic cell transplantation.

## 1. Introduction

Over the past two decades, there has been a remarkable increase in the identification of diseases associated with signal transducer and activator of transcription (STAT) protein defects as well as a much greater understanding of STAT biology [1]. Humans have seven STAT proteins that are key components of the Janus kinase (JAK)-STAT signaling cascade, through which many interferons, cytokines, interleukins, and growth factors signal. STAT proteins are integral parts of many biologic functions, including host immunity, growth, and wound healing, making defects in STAT proteins present with highly heterogenous phenotypes [1,2].

**Figure 1:**
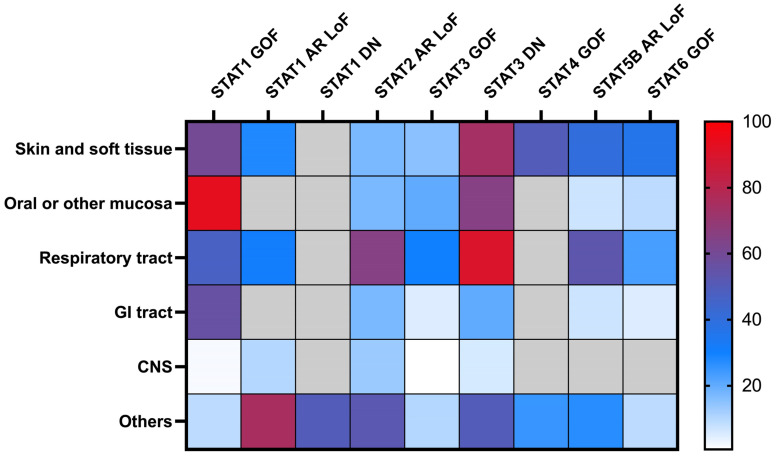
Spectrum of infection susceptibility based on anatomical sites in patients with inborn errors of STATs. STAT2 “GOF” is not included due to the limited number of reported cases, and infection susceptibility is not a primary feature in these patients. The “gray” box indicates a lack of data from published case series, which likely suggests no significantly increased risk beyond that of the general population. GI, gastrointestinal; CNS, central nerve system.

Inborn errors of immunity (IEI) linked to STAT proteins can arise from either loss-of-function (LOF) or gain-of-function (GOF) defects [3]. LOF defects typically result from biallelic deficiency or monoallelic dominant negative (DN) variants, which are inherited in autosomal recessive and dominant patterns, respectively. In contrast, GOF defects are usually associated with monoallelic variants that follow an autosomal dominant inheritance pattern. Clinical manifestations of these defects range from primary immune deficiencies with life-threatening infections to primary immune regulatory disorders with variable autoimmunity and autoinflammation. The pathogens associated with these defects range across bacteria, mycobacteria, viruses, and fungi. In this review, we describe the types of infections associated with the different STAT defects as well as approaches to infection prevention (Table 1 and Figure 1).

## 2. STAT1

STAT1 is essential to host immunity as the major mediator for types I (IFN-α/β), II (IFN-γ) and III (IFN-λ) interferons [4]. In addition, many cytokines, such as IL-2 and IL-6, as well as growth factors, such as platelet-derived growth factor and epithelial growth factor, utilize STAT1 for signaling [2]. Therefore, defects in this pathway can cause significant immune dysregulation. IEI associated with STAT1 defects include biallelic STAT1 deficiency (complete and partial), heterozygous STAT1 deficiency, and STAT1 GOF [3].

### 2.1. Infection Susceptibility

Due to the impaired signaling of both IFN-α/β and IFN-γ, patients with biallelic STAT1 deficiency have high morbidity and mortality in the first year of life from infection and inflammatory diseases, making them more comparable to severe combined immunodeficiency (SCID) than other STAT1 defects. Depending on the genetic defect, both complete and partial STAT1 deficiency have been reported, with more fulminant presentations in those with complete deficiency [5,6,7]. Nontuberculous mycobacteria (NTM) and viral susceptibility are the most pronounced infections in these patients. In a study of 32 patients with STAT1 deficiency, all those who received bacillus Calmette–Guérin (BCG) vaccination developed BCGosis, and those who did not receive the BCG vaccination had high incidences of environmental NTM, with multiple species reported [8]. Several of these cases were fatal or relapsed after initial treatment. Severe viral infections occurred in about two-thirds of the patients. Herpesvirus infections were most common, including herpes simplex virus (HSV), cytomegalovirus (CMV) and varicella zoster (VZV), with many having severe disease such as HSV encephalitis or CMV pneumonitis. Human herpesvirus-6 (HHV-6) was reported to cause hemophagocytic lymphohistiocytosis (HLH). Respiratory viral infections were reported to be more severe, including one patient requiring brincidofovir therapy for disseminated adenovirus. Most patients were already ill before the age at which they could receive live-attenuated viral vaccines and thus had not been vaccinated with measles, mumps, and rubella (MMR) and/or varicella (VZV) vaccines. However, in five patients with complete STAT1 deficiency who received MMR and/or VZV vaccination, all developed vaccine-related adverse reactions, including severe disseminated disease. Outcomes were less severe in those with partial STAT1 deficiency.

Heterozygous *STAT1* DN variants typically cause impaired phosphor–STAT1 dimerization or DNA binding, leading to defective IFN-γ responses but less impaired IFN-α/β-mediated antiviral activity. Compared to biallelic STAT1 deficiency, NTM infections in these patients are typically more localized, such as with osteomyelitis, with a better response to therapy [9,10,11].

**Figure 2:**
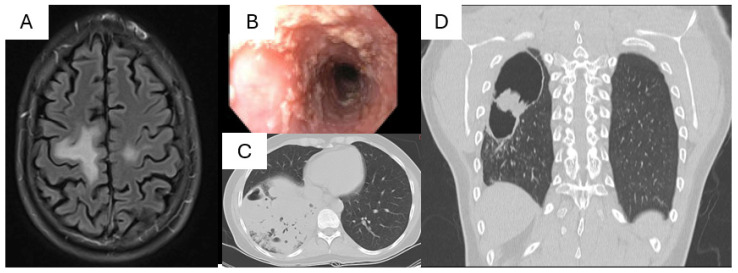
Clinical images of STAT1 GOF and STAT3 HIES: (**A**) brain MRI showing white matter changes due to John Cunningham virus-related progressive multifocal leukoencephalopathy (PML) in a 30-year-old male with STAT1 GOF; (**B**) esophageal candidiasis seen in an endoscopy of a 34-year-old male with STAT1 GOF; (**C**) *S. aureus* lobar pneumonia in a 40-year-old woman with STAT3 HIES; and (**D**) pneumatocele complicated by an aspergilloma in a 24-year-old male with STAT3 HIES.

STAT1 GOF is the most common form of STAT1 defects. It was initially described in patients with chronic mucocutaneous candidiasis (CMC), and CMC remains the most common infection seen in these patients (Figure 2) [12,13]. However, the clinical phenotype and infection susceptibility is much broader, including bacterial, mycobacterial, and viral infections, as was illustrated in a clinical description of 274 patients by Toubiana et al. in 2016 [14]. Almost all patients had a history of mucocutaneous candidiasis, with oral thrush being the most common presentation. Over time, the Candida infections may become increasingly resistant to antifungals, due to the common, and understandable, practice of repeated treatment and frequent prophylaxis with azoles. Less frequent fungal infections include disseminated endemic mycoses [15], such as *Coccidioides* and *Histoplasma*; filamentous mold infection and *Pneumocystis* infections are infrequent.

Recurrent bacterial infections occur in about 75% of patients, with recurrent pneumonias in about half of them. A subset of patients has progressive B cell dysfunction [16], resulting in hypogammaglobulinemia, which would be expected to further increase the risk. About one-third of patients show susceptibly to viral infections, with recurrent HSV and VZV infections being the most frequent. However, serious viral infections can occur, albeit rarely, such as John Cunningham virus-driven progressive multifocal leukoencephalopathy (PML), which can be fatal (Figure 2) [17]. Some of these cases of PML occurred after systemic immunosuppression for autoimmune manifestations, but this is not always present. Although generally there is not an increased susceptibility to respiratory tract infections, including SARS-CoV-2, variable and severe cases of COVID-19 have been described [18,19].

### 2.2. Infection Prevention

For STAT1 deficiency, early consideration of hematopoietic cell transplantation (HCT) is essential for long-term survival [8]. While awaiting HCT, prophylaxis should include anti-NTM therapy, such as azithromycin, if disseminated NTM is not already present; if disseminated NTM is present, patients require combination antibiotics. In addition, prophylaxis should be considered in a similar manner as patients treated for SCID, with prophylaxis against *Pneumocystis jiroveci* pneumonia (PJP), and a consideration of antivirals. For autosomal dominant STAT1 deficiency, prophylaxis for NTM, such as azithromycin, should be provided, assuming that disseminated NTM has been excluded or adequately treated.

Infection prevention for STAT1 GOF depends on the clinical presentation, which is quite variable. Many patients are given antifungal prophylaxis, such as fluconazole, and this should be strongly considered in any patient living in a *Coccidioides* endemic region. However, one initially surprising observation is that JAK inhibition therapy, which is used frequently in STAT1 GOF for autoimmunity, can improve CMC [20,21]. This is thought to be, in part, because the IFN-γ-driven inflammation leads to a loss of mucosal integrity and *Candida* susceptibility [22]. However, care needs to be given to treat patients with disseminated infections prior to the initiation of JAK inhibition, as progressive disease, including *Coccidioides*, has been described [23]. Antiviral prophylaxis should be considered in patients with recurrent HSV or herpes zoster, as well as for those treated with JAK inhibitors due to the increased risk of viral reactivation with those medications [24]. PJP prophylaxis should be considered in patients with significant T lymphopenia. Over time, patients with STAT1 GOF may develop hypogammaglobulinemia; therefore, immunoglobulin replacement therapy (IgRT) should be considered, as well as antibiotics, such as azithromycin, for those with recurrent pulmonary infections, especially in the presence of bronchiectasis [25]. Initial outcomes of HCT in STAT1 GOF were relatively poor, with an overall survival rate of 40% and increased rates of graft rejection [26]. However, as our understanding of IFN-γ-driven inflammation has improved, HCT centers have begun using JAK inhibitors or IFN-γ blocking agents (e.g., emapalumab) as bridge therapies, which appear to be leading to better outcomes [27,28].

## 3. STAT2

STAT2 is ubiquitously expressed but is unique among the STAT family, given its exclusive involvement in signal transduction of type I and III IFNs [29]. STAT2 also plays a negative regulatory role in type I IFN signaling by recruiting USP18 to type I IFN receptor subunit IFNAR2 [30]. Monogenic STAT2 LOF is an autosomal recessive disorder caused by either frameshift or splicing variants, leading to nonsense-mediated RNA decay [31,32,33,34,35,36]. As expected, the phenotype of these patients is predominately viral susceptibility attributed to inadequate IFN-I/III antiviral activity, while they also have hyperinflammation in response to viral infections (even HLH) [34,35], and defects in mitochondrial fission [32,37]. In contrast, STAT2 “GOF” is classified as a type I interferonopathy, characterized by recurrent episodes of severe, multisystemic hyperinflammation, accompanied by neurologic features (e.g., intracranial calcification) [38,39]. Its clinical presentations may resemble the sequelae of intrauterine infection, although no infectious etiology is identified; hence, it is also referred to as Pseudo-TORCH syndrome-3. Although overactive STAT2 could theoretically lead to excessive type I IFN signaling, the variants identified to date (R148W and R148Q) are homozygous, and impair the interaction between STAT2 and USP18 in vitro, leading to unrestrained IFNAR signaling due to a loss of negative regulation [38,39]. Therefore, the term “GOF” in this context describes the phenotype rather than accurately reflecting the molecular mechanism.

### 3.1. Infection Susceptibility

STAT2 LOF patients showed increased susceptibility to a wide range of naturally acquired viral infections affecting various sites, including the skin (e.g., VZV), oral mucosa (e.g., HSV-1), respiratory tract (e.g., influenza, RSV, SARS-CoV-2), GI tract (e.g., rotavirus, norovirus), CNS (e.g., enterovirus, HSV-1), and others (e.g., EBV, CMV, HHV-6). Some of these can be severe, particularly influenza pneumonia, COVID-19 pneumonia, and HSV-1 encephalitis [36,37]. A striking feature in these patients is their extreme vulnerability to diseases caused by live-attenuated viral vaccines [31,32,33,34,35,36], such as MMR, which is also common in other monogenic disorders affecting type I/III IFN signaling [40]. They typically develop disseminated viral disease after vaccination, accompanied by systemic inflammation with features resembling either atypical Kawasaki disease or HLH. Mortality is high in early childhood (~35%), with most patients dying from heart failure in the context of a febrile illness attributed to infection, although a specific microbiologic etiology is often not identified [36]. In contrast, infection susceptibility is not a primary feature of STAT2 “GOF”, although it may occur along the disease course, either as a trigger for subsequent hyperinflammation or in the context of pre-existing critical illness [38]. These patients appear to tolerate live-attenuated vaccines (MMR [38] and BCG [39]) without reported complications.

### 3.2. Infection Prevention

Patients with STAT2 LOF should avoid live-attenuated viral vaccines; however, other inactivated and recombinant vaccines are recommended, including influenza and SARS-CoV-2 vaccination. Some patients have received acyclovir prophylaxis, and/or IgRT (without known baseline hypogammaglobulinemia) [36]. Although the benefits of these interventions cannot be assessed from the limited number of reported patients, acyclovir or valacyclovir are low-risk medications and worth considering. None of these patients have undergone HCT; viral susceptibility from impaired type I/III IFN signaling in the non-hematopoietic compartment would not be corrected by HCT.

For STAT2 “GOF”, early treatment of infections may help to mitigate the risk of subsequent severe hyperinflammation. A better disease-modifying strategy is needed, which could also reduce the risk of secondary infection during critical illness, as responses to steroids and JAK inhibitors have been inadequate [38]. Anifrolumab, a monoclonal antibody against type I IFN receptors, could be a potential option. It led to clinical and biochemical improvement in a patient with type I interferonopathy due to a homozygous *DNASE2* LOF variant [41], although it is important to be mindful of the associated risk of certain viral infections (i.e., respiratory virus and herpes zoster) when using this therapy [42].

## 4. STAT3

STAT3 is expressed widely in tissue types and involved in the signaling of the IL-6 family, IL-10 family, IL-21, IL-27, as well as in growth factors. Dominant negative variants in STAT3 (STAT3 DN) were described in 2007 as the most common cause of autosomal dominant hyper-IgE (Job’s) syndrome (STAT3 HIES) [43,44]. More recently, STAT3 GOF disease has been described primarily in patients with autoimmunity and lymphoproliferation [45,46].

### 4.1. Infection Susceptibility

STAT3 HIES is a multi-system disorder characterized by eczematoid dermatitis, recurrent skin and lung infections, retained primary teeth, skeletal and joint abnormalities, and vasculopathy [47,48,49]. *Staphylococcus aureus* causes cutaneous abscesses and pneumonia, usually starting in the first few years of life (Figure 2). *S. aureus* colonization also drives the eczema, and suppression through antiseptics or antibiotics can lead to significant improvements. Specific antibody production is often poor in STAT3 HIES and *Streptococcus pneumoniae* and *Haemophilus influenzae* cause ear and sinus infections as well as pneumonias. Healing from pneumonias is abnormal; frequently, the patients are left with pneumatoceles and/or bronchiectasis (Figure 2) [50]. Once the lung parenchyma is disrupted in this way, the infecting pulmonary pathogens change and patients may develop chronic infections with Gram negatives such as *Pseudomonas aeruginosa*, molds such as *Aspergillus* species, and NTM [51,52,53].

Fungal infections are common in STAT3 HIES [51]. Chronic mucocutaneous candidiasis affects about 85% of patients, typically as oral thrush or nail infections, and is related to defective STAT3/Th17 immunity [54]. PJP is infrequent but can occur during infancy, and much more rarely in adults [55]. Pulmonary aspergillosis and other molds can present in different forms, including fungal balls in pneumatoceles (Figure 2), chronic airway disease complicating bronchiectasis, and allergic bronchopulmonary mycoses (ABPM) [52]. Although invasive disease with dissemination beyond the airways is rare, there is often local invasion that can lead to significant bronchiectasis. *Fusarium* species infrequently cause chronic skin and soft tissue disease that can be difficult to treat due to its frequently extensive antifungal resistance [56]. Individuals with STAT3 HIES are also susceptible to endemic mycoses. Disseminated histoplasmosis frequently leads to significant bowel disease that can mimic inflammatory bowel disease. Cryptococcus and Coccidioides can cause meningitis [57].

STAT3 HIES typically does not result in more severe viral infections. However, related to the low-memory T lymphocytes, the reactivation of chronic viruses can occur [58]. This can be subclinical, such as with EBV viremia, but there is a relatively high incidence of zoster, manifesting in a similar manner to those who are immunocompetent, with a single dermatome. SARS-CoV-2 has not caused particularly severe disease in this population; however, respiratory viruses, in general, can lead to exacerbations for those with bronchiectasis.

Haploinsufficiency has emerged as a novel mechanism of STAT3 defects. One patient had a de novo heterozygous splice-site variant (c.1140-3C>G), which disrupted splicing and resulted in a premature stop codon (p.S381*) with nonsense-mediated decay. His clinical phenotype was notable due to invasive sino–orbital–cerebral aspergillosis, which developed around age 37 and led to his death. Otherwise, he did not have the connective tissue abnormalities typically seen in patients with *STAT3* DN variants [59]. Similarly, two other patients had unremarkable histories until the development of disseminated coccidioidomycosis, which was eventually fatal due to central nerve system involvement [60].

STAT3 GOF is characterized primary by autoimmunity and lymphoproliferation, but recurrent and/or chronic infections do occur in about 75% of patients [61]. Sinopulmonary bacterial infections are most common and are frequently found in patients who have associated hypogammaglobulinemia. Viral and fungal infections are reported as well, but it is unclear in how much of the infection susceptibility relates to the immunosuppressants that the majority of patients receive.

### 4.2. Infection Prevention

Infection prevention in STAT3 HIES is geared towards preventing pneumonias to hopefully minimize progression to lung pneumatoceles and bronchiectasis, which drive much of the morbidity and mortality in this disease [47]. Trimethoprim/sulfamethoxazole (TMP/SMX), dosed at 5–6 mg/kg/day of the TMP component, divided twice daily (maximum dose 160 mg TMP component), is most frequently used to prevent *S. aureus* pneumonias as well as to help control *S. aureus* of the skin leading to eczema and abscesses. Eczematoid dermatitis can also be improved frequently with antiseptics, such as diluted bleach baths and/or washing with chlorhexidine, to decrease the *S. aureus* colonization. In recent years, dupilumab has increasingly been used for STAT3 HIES with remarkable improvement of the dermatitis, which also seems to be lowering the rate of skin abscesses [62,63,64].

If bronchiectasis is present and leading to recurrent exacerbations, azithromycin may be considered, although the exclusion of pulmonary NTM through sputum cultures should ideally be performed first [65]. Airway clearance is also advised such as with hypertonic saline nebulization or airway clearance techniques. Antifungal prophylaxes are considered for those with frequent CMC infections, typically with fluconazole. For those with pneumatoceles that do not have mold infection/colonization, itraconazole can be considered to ideally prevent pulmonary mold infection. For those with pulmonary mold infections, such as with *Aspergillus* spp., typically long-term, potentially lifelong, mold therapy is considered, such as with a triazole (e.g., posaconazole, isavuconazole). Although voriconazole may be used to treat the mold infections, long-term use has been associated with photosensitivity and skin cancers [66,67], as well as fluorosis [68]. For those in Coccidioides-endemic regions, antifungal prophylaxes, such as fluconazole, should be provided due to the severity of the brain infections. Counseling may also be provided to avoid high-risk settings for fungal infections such as mulching or hayrides. Although serum IgG levels are typically normal, specific antibody production is often poor; therefore, IgRT can be considered and was shown to decrease pneumonias in a French retrospective study [51].

Routine vaccinations, including influenza and SARS-CoV-2 vaccinations, should be provided; however, increased large local reactions were anecdotally reported with the pneumococcal polysaccharide vaccine [69]; therefore, the use of protein-conjugated vaccines solely for pneumococcus may be prudent.

The role of HCT in STAT3 HIES remains unclear. As STAT3 is expressed throughout tissue types, leading to a multi-system syndrome, HCT will not be curative. However, eczematoid dermatitis and recurrent infections have been improved with HCT and can be considered in patients with continued breakthrough infections, especially pulmonary infections that occur despite optimal supportive care [47,70].

In STAT3 GOF, the infections are variable and frequently related to either recurrent sinopulmonary bacterial infections with hypogammaglobulinemia or viral infections associated with immunosuppression and/or lymphopenia. IgRT is frequently indicated for those with hypogammaglobulinemia to prevent bacterial infections [61]. If bronchiectasis is present, other measures to prevent infections may be utilized such as azithromycin (if there is no pulmonary NTM) and airway clearance techniques. JAK inhibitors are increasingly used to treat autoimmunity and inflammation in STAT3 GOF and can increase the risk of viral reactivation, leading to infections such as zoster [21,24]. Therefore, acyclovir or valacyclovir should be considered. PJP prophylaxis can be considered in a case-by-case manner, depending on the concurrent immunosuppression. The role of HCT is unclear for STAT3 GOF, with relatively worse outcomes in initial attempts; this is likely related to those with transplants having more severe end-organ disease [61]. With more experience, HCT outcomes are expected to improve.

## 5. STAT4

STAT4 is expressed in both human skin and leukocytes [71,72]. It is essential for mediating the transcriptional response to IL-12 but can also be activated by other cytokines (e.g., type I IFNs and IL-23) [72]. Although single-nucleotide polymorphisms (SNPs) in *STAT4* have been linked to various immune-mediated disorders by genome-wide association studies (GWAS), the only monogenic disorder identified to date is autosomal dominant STAT4 GOF with a phenotype of disabling pansclerotic morphea of childhood, reported in four patients [73].

### 5.1. Infection Susceptibility

Of the four patients, two (i.e., patient 2 and 3) had recurrent infections [73]. Both experienced cellulitis associated with non-healing sclerotic skin ulcers. Microbiologic evaluation was unavailable for patient 2; his case was further complicated by squamous cell carcinoma. He ultimately underwent right above-the-knee and bilateral through-humerus amputations due to persistent skin lesions with frequent cellulitis and bleeding episodes. For patient 3, cultures from debrided skin ulcers grew *Serratia marcescens* and *Enterococcus faecalis*. In addition, a lymph node biopsy obtained during the evaluation of interstitial lung changes suggested CMV infection, and his death occurred at age 31 due to an unspecified infection. These observations suggest that infectious complications in STAT4 GOF are primarily related to the impaired skin barrier, although immune dysregulation likely contributes to its complexity, as cytopenias and hypogammaglobulinemia were seen in some patients.

### 5.2. Infection Prevention

The primary skin fibroblasts for three STAT4 GOF variants exhibited a hyperinflammatory phenotype, which was mitigated by ruxolitinib treatment in vitro. These observations led to the initiation of ruxolitinib in two patients (i.e., patient 1 and 2) with notable improvements in skin lesions, cytopenias, and several biochemical markers. Of note, no further infectious complications were reported in patient 2 after starting ruxolitinib. Given the progressive course of disease with the limited success of supportive wound care, antibiotics treatment, and conventional immunosuppressive/modulatory agents, the early introduction of disease-modifying therapy with a JAK inhibitor should be considered [74], which may prevent the development of further infectious complications.

## 6. STAT5B

STAT5B is broadly expressed across different tissues and mediates signal transduction triggered by a range of cytokines (e.g., IL-2 family), hematopoietic growth factors (e.g., Flt3 ligand, erythropoietin), and growth hormones [75]. Two forms of germline LOF monogenic disorders have been reported: growth hormone insensitivity with immune dysregulation type 1 (autosomal recessive) and type 2 (autosomal dominant). The hallmark of these patients is growth failure, characterized by short stature, delayed bone age, and delayed puberty. Several types of infections have been described in most cases of the former condition [76,77,78,79,80,81,82,83,84,85,86]. In contrast, the latter condition does not appear to be associated with an increased risk of infections; however, only about 12 cases have been reported to date, with a focus on growth failure [87,88]. Additionally, somatic *STAT5B* GOF variants in the hematopoietic lineage have been linked to T-cell clonal diseases [89,90], primarily large granular lymphocyte leukemia. However, two patients with non-clonal eosinophilia, urticaria, dermatitis, and diarrhea were also reported [91].

### 6.1. Infection Susceptibility

Recurrent skin and respiratory tract infections were observed in nearly half of the cases with autosomal recessive STAT5B LOF. Notably, six patients had a history of severe varicella skin infection during childhood, with three described as “hemorrhagic” [76,78,79,80,82]. Moreover, one case was complicated by ocular involvement (i.e., keratitis and uveitis) leading to progressive loss of visual acuity [79], while another patient experienced several episodes of herpes zoster later in life [82]. Otherwise, bacterial or fungal skin infections have not been a significant concern. In terms of respiratory infections, viral etiologies (e.g., rhinovirus, adenovirus, and influenza) were identified in several cases [92]; however, two patients developed PJP. Both occurred in the context of poorly controlled lymphoid interstitial pneumonia [76,82], although the details of PJP onset relative to concurrent immunosuppressive therapy are not available. In addition, two patients had EBV viremia [92].

### 6.2. Infection Prevention

The immune defects in patients with autosomal recessive STAT5B LOF are variable. T-cell lymphopenia (both CD4^+^ and CD8^+^) and NK cell defects with impaired terminal maturation as well as lytic function likely contribute to the increased risk of viral and opportunistic infections. However, the concurrent use of steroids and immunosuppressants may also play a role [83,92]. No disease-modifying therapy or allogeneic stem cell transplantation has been reported in these patients. Early evaluation of any infectious complications in an appropriate clinical setting is always recommended. The management currently relies on appropriate antimicrobials; rituximab has been used for EBV viremia treatment in two patients [92]. Prophylaxes against PJP in the context of CD4^+^ lymphopenia and/or prolonged steroid use should be considered.

## 7. STAT6

STAT6 is expressed in both immune and non-immune cells (e.g., epithelial cells and fibroblasts) [93]. It plays a crucial role in transducing signals from IL-4 and IL-13 cytokines, which are key regulators in type 2 immunity. GWAS have linked *STAT6* SNPs with various human atopic disorders [94]. Recently, a new primary atopic disorder caused by germline heterozygous *STAT6* GOF variants was reported by several research groups [95,96,97,98,99,100]. This condition, now also known as hyper-IgE syndrome 6, autosomal dominant, with recurrent infections, is characterized by severe multi-system atopic manifestations with a significant risk of life-threatening anaphylaxis [94]. In addition, B-cell lymphoma was described in one patient [99].

### 7.1. Infection Susceptibility

The most common infections described in these patients are recurrent skin infections, almost always occurring in the setting of severe, poorly controlled atopic dermatitis. Associated pathogens include bacteria (e.g., *S. aureus*, group A/G *Streptococcus*), fungi (e.g., *Candida spp.*), and viruses (e.g., herpes simplex virus, molluscum contagiosum virus) [95,97,100]. Complications ranged from skin/soft tissue abscesses to more severe conditions, e.g., septic arthritis, and bacteremia. Additionally, frequent respiratory infections were reported in a subset of these patients, often of viral origin and typically identified during evaluations of asthma exacerbations [95]. One patient also experienced recurrent oropharyngeal and esophageal candidiasis, which responded to fluconazole therapy. The candidiasis occurred in a setting of uncontrolled eosinophilic esophagitis despite an elimination diet, swallowed steroids, and subsequent oral steroids [95]. Overall, infections in STAT6 GOF are usually secondary and associated with uncontrolled atopic manifestations, which may also underscore the complex interplay between type 2 immunity, barrier dysfunction, and susceptibility to infections in STAT6 GOF.

### 7.2. Infection Prevention

The germline GOF variants studied to date showed increased STAT6 transcriptional activity, resulting in the upregulation of key downstream targets, including IL-4Rα, as observed in primary cells (e.g., CD4^+^ T and B cells) from patients. Furthermore, these patients were found to have higher levels of type 2 cytokines (i.e., IL-4, IL-5, and IL-13) in both serum and by intracellular staining [95]. These findings provide a rationale for the therapeutic use of dupilumab, a monoclonal antibody targeting IL-4Rα, which is approved for multiple atopic disorders such as atopic dermatitis, asthma, and eosinophilic esophagitis. Among the several patients with STAT6 GOF who received dupilumab therapy, there was a significant improvement in atopic dermatitis control, with no recurrence of skin infections reported afterwards [95,97,100]. Collectively, these observations suggest that, in addition to antimicrobial therapy when indicated, controlling type 2 inflammation is key to preventing further infections in STAT6 GOF.

## 8. Conclusions

Inborn errors of STATs are linked to a broad spectrum of infectious complications due to the critical role of JAK-STAT signaling in both hematopoietic and non-hematopoietic compartments. As our clinical experience accumulates and the underlying pathophysiology becomes clearer, the susceptibility to certain infections in each defect is increasingly recognized (Table 1 and Figure 1). Infection prevention can be achieved through the use of selected antimicrobial prophylaxis based on the infection susceptibility, as well as through optimized disease management with either targeted therapies or HCT, as appropriate.

## Figures and Tables

**Table 1:** Pathogen-based infection susceptibility in patients with STAT defects.

Defects ^1^	Bacteria: *S. aureus*	Bacteria: Pyogenic	Mycobacteria	Herpes Viruses	Respiratory Viruses	Vaccine Acquired Virus	Candida	Molds	Endemic Fungi
AR STAT1 LOF	+ ^5^	+	+++	++	++	++	+	+	-
AD STAT1 LOF	-	-	+++	-	-	-	-	-	-
STAT1 GOF	+	++	+	++	+	+	+++	+	++
STAT2 LOF	-	-	-	++	++	+++	-	-	-
STAT3 DN	+++	++	+	+	-	-	+++	++	++
STAT3 GOF ^2^	+	++	+	+	+	-	-	+	-
STAT4 GOF ^3^	+	+	-	-	-	-	-	-	-
STAT5B LOF	+	+	-	++	+	-	-	-	-
STAT6 GOF ^4^	++	+	-	+	-	-	+	-	-

^1^ STAT2 GOF and STAT3 haploinsufficiency are not included in the table as very few patients have been reported; ^2^ Many of the infections in STAT3 GOF are likely related to the frequent bronchiectasis and immunosuppressants use for autoimmunity; ^3^ Most infections are likely related to the impaired skin barrier; ^4^ STAT6 GOF is also associated with viral skin infections such as molluscum contagiosum, typically in the setting of impaired skin barrier from uncontrolled atopic dermatitis; ^5^ The risk of infections is categorized as follows: - (not significantly increased), + (mildly increased), ++ (moderately increased), and +++ (significantly increased) in comparison to the general population.

## Data Availability

No new data were created or analyzed in this study. Data sharing is not applicable to this article.

## Clinical Trial Registration

Clinical images in Figure 2 were obtained from patients enrolled in natural history protocols at the National Institute of Allergy and Infectious Diseases, National Institutes of Health (NCT00006150, NCT00001355).
